# Assessing variability and uncertainty in orthopedic randomized controlled trials

**DOI:** 10.1080/17453674.2020.1755932

**Published:** 2020-04-22

**Authors:** Lauri Raittio, Aleksi Reito

**Affiliations:** a Tampere University, Faculty of Medicine and Health Technology, Tampere;; b Department of Orthopaedics and Traumatology, Tampere University Hospital, Tampere, Finland

## Abstract

Background and purpose — Low statistical power remains endemic in clinical medicine including orthopedics and manifests as high uncertainty and wide confidence intervals (CI). We evaluated the reporting and correspondence between power calculation and observed data on key parameters of variability and uncertainty in orthopedic randomized controlled trials (RCTs).

Material and methods — RCTs with 1:1 allocation published in 8 major orthopedic journals between 2016 and 2017 with one continuous primary outcome were included in the review. The components of power calculation and observed standard deviation (SD), mean difference (MD), and confidence interval (CI) of MD between groups were assessed for primary outcome.

Results — 160 RCTs were included, of which 93 (58%) and 138 (86%) studies reported the estimated SD and MD in the power calculation, respectively. The median ratio of the estimated SD and SDs observed in the data was 1.0 (IQR –0.76 to 1.32) for 69 (43%) studies. Only 31 of 138 studies reported the CI of MD in primary outcome. In 42% of the negative studies, the estimated MD was included in the CI of the observed MD.

Interpretation — The key parameters of data variability, both in power analyses and in final study results, were poorly reported. Low power in orthopedics may result from too high an estimated effect size due to an overoptimistic estimate of MD between study groups. In almost half of the studies, overlap of the CI of the observed MD and estimated MD suggested that the reported results of these studies were inconclusive.

Adequate statistical power is the cornerstone of reproducible and high-quality clinical research. High statistical power is needed to increase the likelihood that a study will detect an effect when there is an effect to be detected. According to the CONSORT statement (Schulz et al. 2010), power calculations are based on the estimated mean difference (MD_est_) between compared groups, the estimated standard deviation (SD_est_) or variability of the outcome at a particular point in time, and the chosen level of error, namely, type 1 and 2 errors. A complement of type 2 error is statistical power.

Despite the increasing use of power calculations, low power among RCTs to find small and medium effect sizes still remains endemic in clinical medicine, including orthopedics (Button et al. 2013, Abdullah et al. 2015, Sabharwal et al. 2015, Szucs and Ioannidis 2017, Reito et al. 2020). In studies using a priori power analysis, low power may arise from overestimated mean difference (MD), from underestimated standard deviation (SD), or from both (Vickers 2003, Cook et al. 2018). In many orthopedic RCTs, a patient-level minimal clinically important difference (MCID) is currently the basis of the group-level MD_est_ used in power calculations. Usually, the MD_est_ used in power calculations represents the clinically relevant difference valued by the investigators (Ostelo et al. 2008, de Vet and Terwee 2010, Angst et al. 2017, Jayadevappa et al. 2017, Dabija and Jain 2019). In this study we use the terms “MD_est_” and “MCID” interchangeably.

Small sample sizes will yield high uncertainty of the outcome variable, which may, in turn, manifest as wide confidence intervals (CIs) (Anderson 2019). The mainstay in the interpretation of negative trials is to declare no statistically significant difference or “no difference” between the study groups if the CI of MD (CI_MD_) between groups includes equivalence in means, i.e., zero difference. A more appropriate interpretation would be to interpret the CI_MD_ to see which values for group difference are excluded by the data based on the chosen confidence level (Gelman and Greenland 2019).

In this systematic review, we investigated (1) the reporting of the key parameters of variability and uncertainty; (2) the correspondence of the SD_est_ of the primary outcome used in the power analysis to that actually observed in the study population; (3) the overlap of the MD_est_ between groups to the CI_MD_ in the primary outcome between study groups, and (4) the difference in sample size and estimated effect size in studies with and without overlap in MD_est_ and CI_MD_ in orthopedic RCTs published in 8 journals in the years 2016 and 2017.

## Material and methods

### Study selection

We reviewed 8 journals focused on clinical orthopedic research, namely the Journal of Bone and Joint Surgery; Clinical Orthopaedics and Related Research; the Bone and Joint Journal; the American Journal of Sports Medicine; Arthroscopy; the Journal of Arthroplasty; Knee Surgery, Sports Traumatology, Arthroscopy; and Acta Orthopaedica.

The electronic table of contents from the 2016 and 2017 volumes of each of the 8 journals were searched issue by issue in chronological order to identify any RCTs. All studies that claimed to be a 1:1 RCT were included in the analysis.

### Data extraction

All selected studies were examined in detail. The use of power analysis and the type of primary outcome (continuous, binary, noninferiority, other) used in the studies was recorded. We used the primary outcome and the power outcome in this study interchangeably. If continuous primary outcome was used in the power analyses, we recorded the MD_est_ and SD_est_ used to derive the sample size estimate. The number of patients available, means, and estimate of variability (SD or standard error, SE) for both study groups (i.e., SD_1_, SD_2_) at the pre-specified or at the latest follow-up time point when the results were reported was recorded. If these were not reported, we assessed whether the authors had reported CIs for the primary outcome in the study groups (CI_1_ and CI_2_). In cases where the SDs of primary outcome (SD_1_ and SD_2_) for the study groups were not reported, they were calculated from the SEs (SE1 and SE2) or CIs (CI_1_ and CI_2_) if reported. For all studies where the SDs of primary outcome for the study group were reported or calculated, we also calculated the pooled SD (SD_pooled_) of the primary outcome in the study participants. This was calculated as described in the Cochrane handbook (Higgins and Green 2011). Assuming sample sizes were reported, SD_pooled_ was calculated from the CI_MD_ if the SDs for the study groups were not available. Finally, we assessed whether the authors had reported the CI for the MD between the groups (CI_MD_). However, if the observed CI_MD_ was not found, it was calculated from the SD_pooled_, assuming the authors had reported the sample size and the study group means for the primary outcome.

### Data assessment

**Figure 1.**
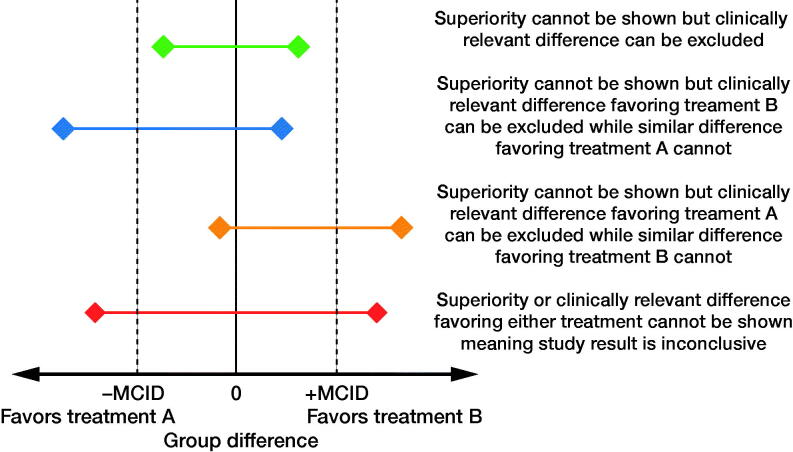
Interpretation of studies in which similarity between study groups cannot be rejected, i.e., “negative studies.”

The ratio of the observed and estimated SDs (SD_1_/SD_est_, SD_2_/SD_est_, and SD_pooled_/SD_est_) was calculated for each study. The median, inter-quartile range (IQR), and geometric mean (SD) values for these ratios were reported. For each study, the overlap of MD_est_ with regard to the upper and lower boundary of CI_MD_ was investigated. This was basically a unidirectional analysis, i.e., we checked whether the higher of the absolute values of the upper and lower limit of CI_MD_ was smaller or higher than MD_est_ used in power calculation of the study. Of the “negative” RCTs, i.e., those studies that reported statistically not-significant results, the proportion of studies with and without this bidirectional overlap was reported. In other words, we checked whether the lower limit of CI_MD_ was higher or lower than the negative value of MD_est_ or the upper limit of CI_MD_ was higher or lower than the positive value of MD_est_. In 3 studies CI_MD_ was not reported but authors declared significant or nonsignificant results referring to some p-value, and our calculation showed marginal compatibility of data with zero effect size, e.g. (CI –0.16 to 1.72). In these 3 studies, we classified the results to positive and negative groups using the classification of the authors. In the optimal situation, for “negative” studies, CI_MD_ excludes both negative and positive MD_est_ (Figure 1). The estimated effect size in each study was calculated by dividing MD_est_ by SD_est_ and the mean estimated effect sizes and sample sizes were compared between studies with and without overlap between MD_est_ and CI_MD_. In addition, sample sizes were compared using the Mann–Whitney U-test.

## Results

**Figure 2.**
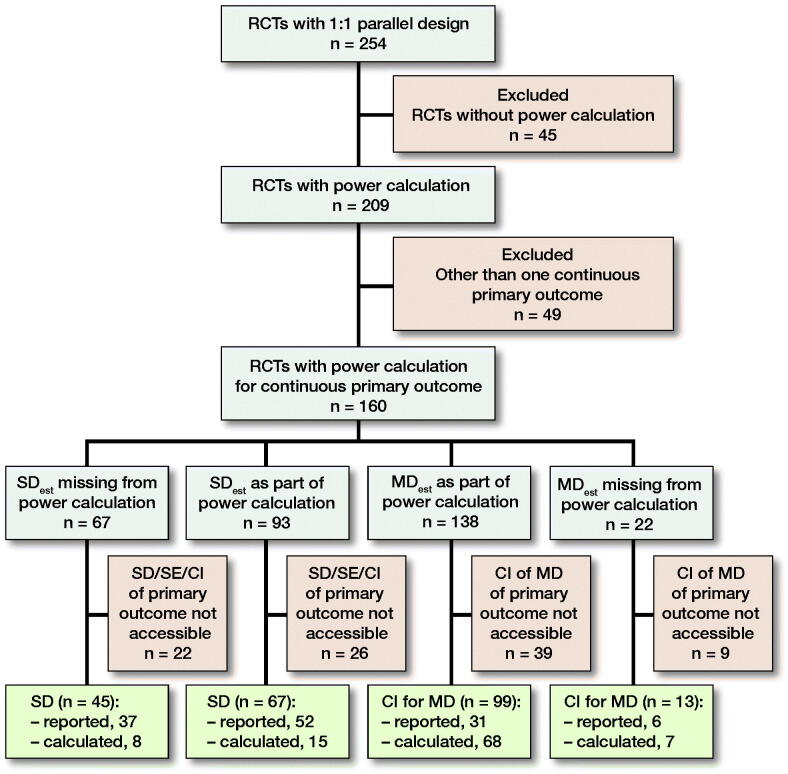
Flow chart of study selection.

Of the 254 RCTs identified in our study, 209 studies (82%) employed a priori power analysis. The primary outcome was binary in 26 (12%), several in 6 (3%), not reported in 2 (1%), and generic in 7 (3%) of these studies. 8 studies (4%) had a noninferiority study setting. In total, 160 (77%) studies had a continuous primary outcome and were included in the analysis (Figure 2).

### Reporting of the key parameters of variability and uncertainty

SD_est_ as a part of the power calculation was reported in 93 (58%) studies (Figure 2). Observed SDs (SD_1_ and SD_2_) of the outcome in the study groups were reported in 89 (57%) studies (Table 1). The rate of reporting SD_1_ and SD_2_ was comparable whether or not power calculation consisted of SD_est_ (Figure 2). Both estimated and observed SDs were reported in 52 (33%) studies. In addition, the observed SDs in the study groups were calculated from the observed SEs or CIs in 15 (14%) of the studies that also reported SD_est_ (Figure 2). A quarter (26/93) of the studies did not report any variability parameter of primary outcome data when SD_est_ was presented (Figure 2). The MD_est_ of the primary outcome in the power calculation was found in 138 studies (86%). Of these, 31 (19%) reported CI_MD_ and in a further 68 (49%) studies they could be calculated from the means and pooled SD, resulting in a total of 99 (72%) studies.

**Table 1.** Reporting of power calculations and study results among 160 orthopedic RCTs

Factor	n (%)
Power analysis	160
Estimated SD reported	93 (58)
Estimated MD reported	138 (86)
Observed variability of primary outcome measure	
SDs of primary outcome for study groups	89 (57)
SEs for study groups	4 (3)
CIs for study groups	19 (12)
CI for mean difference	3 (2)
Data on mean difference between groups	
Reported CI for mean difference	37 (23)
Calculated CI for mean difference	75 (47)

### Correspondence of estimated and observed variability

The pooled SD (SD_pooled_) for the primary outcome variable was calculated for 62 studies in which SD_est_ was also available. The median value for the ratio of pooled observed SD to estimated SD (SD_pooled_/SD_est_) was 1.0 (IQR: 0.76–1.32). The geometric mean value of SD_pooled_/SD_est_ was 1.01 (Table 2).

**Table 2.** Correspondence of estimated and observed variability in the primary outcome, pooled, and in the two study groups, respectively

Measure	Median (IQR)	Geometric mean (SD)
Ratio of observed and estimated pooled SD	1.00 (0.76–1.32)	1.01 (1.62)
Ratio of SD1/SDest	1.03 (0.73–1.43)	1.00 (1.76)
Ratio of SD2/SDest	0.96 (0.74–1.20)	0.96 (1.74)

SD = standard deviation of primary outcome.

### Overlap of the estimated difference and confidence interval of mean difference between groups

**Figure 3.**
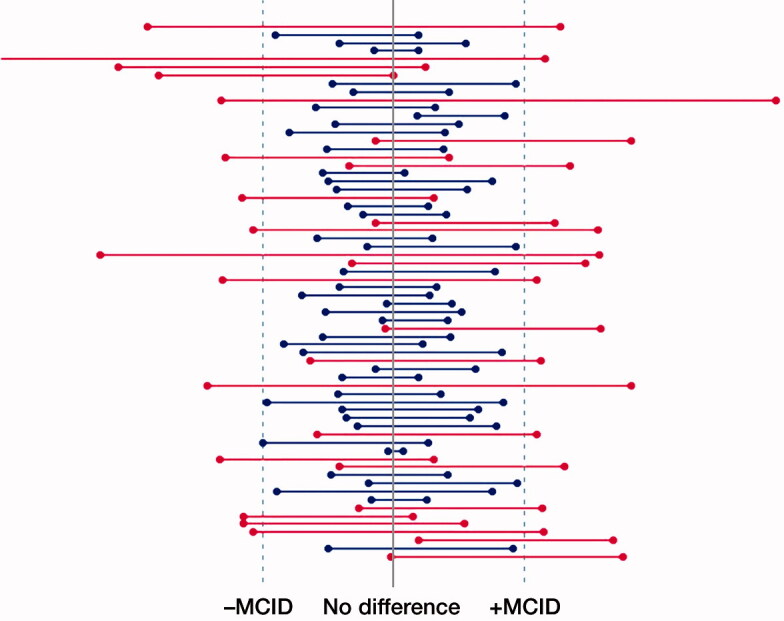
Relative confidence intervals for 66 negative studies, i.e., not reporting a difference, which reported CI_MD_ or in which CI_MD_ was calculated. Blue interval limits highlight studies in which MD_est_ in both directions could be excluded, whereas red indicates studies in which MD_est_ could not be excluded, thus indicating inconclusive studies.

In those studies that had CI_MD_ available, 66 had reported a negative outcome (statistically not-significant finding). Of these, the MD_est_ did not belong to the observed CI_MD_ between groups for the primary outcome at the last or pre-specified follow-up time point in 38 (58%) studies. In other words, 42% of the negative studies could not exclude a clinically meaningful mean difference sized MD_est_ between groups. Figure 3 illustrates the CI_MD_ of these negative studies corresponding to the positive and negative values of MD_est_ chosen in the power calculations (66 studies).

### ^Sample size and estimated effect size in studies with and without overlap in the estimated difference and confidence interval of mean difference^

The median estimated effect size in the power calculation was 0.75 (IQR 0.60–1.0) for those studies that presented both SD_est_ and MD_est_ in the power calculation (Table 3). The mean estimated effect size was greater (0.84 versus 0.79, MD = 0.05, 95% CI –0.7 to 0.26) in negative studies in which the estimated MD_est_ was not included in the CI_MD_. The median sample size in negative studies in which MD was included in the CI_MD_ was 53 (IQR 43–62). In studies where MD_est_ was not included, the median sample size was 86 (IQR 63–115). The groups had a difference in ranks when sample sizes were compared (p = 0.01).

**Table 3.** Median effect sizes in power calculation in all studies and studies divided by the belonging of the mean difference (MD) estimate to the observed confidence interval (CI) of difference in means

Measure	n/N (%)	Median (IQR)	Mean (SD)
Effect size in power calculation of all studies that estimated SD and MD	93/160 (58)	0.75 (0.60–1.0)	0.79 (0.30)
Among “negative” studies, MD estimate did **not** belong in the CI and corresponding estimated effect size	38/66 (58)	0.67 (0.56–0.92)	0.79 (0.39)
Among “negative” studies, MD estimate did belong in the CI and corresponding estimated effect size	28/66 (42)	0.83 (0.60–1.08)	0.84 (0.32)

## Discussion

The rationale for our study was to investigate the etiology of the suggested low power in orthopedic RCTs and the subsequent consequences, namely, are unreasonably small variability estimates to blame for low power and is the uncertainty of the primary outcome measured affected by small sample sizes? Assuming that average statistical power was low among orthopedic RCTs, we hypothesized that there would be poor correspondence in the estimated and observed variability of the primary outcome. In addition, the overlap of MD_est_ and 95% CI_MD_ was investigated in the primary outcome. Therefore, we addressed the correspondence of the estimated variability of the primary outcome in the study population to that actually observed. Also, we compared the MD_est_ of the primary outcome with the observed CI_MD_ for the between group difference among orthopedic RCTs recently published in 8 major scientific journals. We found good correspondence between the estimated and observed SDs based on median values. It is matter of great concern that in almost half of the RCTs there were major deficits in the reporting of the main outcome variables and that a clinically relevant difference between groups could not be excluded based on the CI of the mean difference in primary outcome variable.

Orthopedic researchers have widely incorporated the power calculation in current studies and the power calculation was performed in 160 studies using 1 continuous primary outcome. However, SD_est_ was reported in only 58% of studies, whereas MD_est_ was reported in 86% of all studies reporting power calculation. The SD_est_ and the MD_est_ of the primary outcome are mandatory in power calculation and are of the utmost importance in evaluating the reasonableness of the sample size. Moreover, a quarter (28%) of the studies that presented SD_est_ and MD_est_, in accordance with CONSORT guidelines for power calculation did not report the observed SD, SE, or CI of the primary outcome in the study groups in numerical format. The uncertainty of the investigated effect of the intervention in the RCT can be addressed only by these measures of variability of the mean difference. These missing values of the reported power calculations are in line with the situation a decade ago in the major clinical medicine journals (Charles et al. 2009). Finally, it was also a concern to find that only one-fifth of studies reported the confidence intervals for mean difference value, which is in line with the situation in orthopedics a decade ago (Vavken et al. 2009). In half the studies the CI_MD_ could be calculated, but readers cannot be expected to perform such a calculation. Similar issues in reporting of results in medical literature were noticed by Altman (1980b), who concluded that bad scientific experiments are unethical, from which poor statistical methods and reporting of results are not detached (Altman 1980a). Moreover, praise for reporting CIs instead of solely relying on p-values was expressed long ago by the International Committee of Medical Journal Editors (1988).

Among the studies included in this review, there was good correspondence based on median ratio values between estimated SD and observed SD in the primary outcome, which is contrary to a non-orthopedic review in which the estimated SDs tended to be smaller compared with that actually observed in the study population (Vickers 2003). After all, the estimates of SD and MD in the power calculation are estimates of unknown parameters. Simulation studies show that only a small amount of knowledge of the SD in the population is collected after a total of 70 patients using continuous variables (Teare et al. 2014) and most orthopedic trials include at least this number of patients, and thus yield well-established SD estimates of the population to be used in power analyses.

Inferences made solely on the p-value or nominal significance should be treated with skepticism (Altman 2005). Instead, the CI_MD_ can be used to convey important information about plausible effect sizes, especially in the case of negative trials. In an optimal situation, the MD_est_ in power calculation or another estimate of clinically relevant MD size of difference does not overlap with the CI_MD_ of the primary outcome measure when interpreting negative trials. In almost half of negative studies, the MD_est_ did belong to the observed CI_MD_. Thus, a clinically relevant difference or MCID was excludable with a 0.95 confidence in less than half of the negative studies. A universal misinterpretation of a finding without nominal statistical significance is to declare that there is no difference between groups, suggesting an equivalence. Failure to reject the null hypothesis does not indicate the groups are equal (Altman and Bland 1995) and it should be stated that superiority cannot be established, and results should be interpreted based on the CI_MD_. The CI_MD_ shows which values can be rejected at the chosen error level. If the MCID is included in the CI_MD_, little can be interpreted from the study because the result is inconclusive.

High estimated effect size yields a lower sample size and eventually a point estimate with high uncertainty, i.e., wide CI_MD_. This is the major problem in orthopedic science because wide CIs give very poor inference chances for our studies. It is of course important to remember that increasing sample size always has implications in ethical, pragmatic, and financial aspects. However, little is known about why sample sizes remain low in orthopedic studies. Based on our results, we postulate that since there was good correspondence with SDs, we assume that high effect size estimates are partly due to optimistically high estimates of MD in power calculations. However, it should be noted that greater sample size yields narrower CI_MD_, but, holding constant the alpha and the beta error levels in power calculation, the MD_est_ would be smaller, and results would still often include MD_est_ size of difference in the CI_MD_. The distribution of effect sizes in over 11,000 meta-analyses and their respective RCTs in the Cochrane database shows that almost all effect sizes are small or moderate in size (Lamberink et al. 2018). If the sample size were to be larger than currently seen, it would be able to exclude not only spuriously high estimates of MD, but more realistic ones. Due to these aforementioned issues of the targeted estimate of MD used in power calculations, it has been proposed to view MD_est_ as a context-specific difference “one would not like to miss” (Senn 2014) or to use estimated width of CI for primary outcome instead of MD_est_ as a basis for sample size calculation (Rothman and Greenland 2018).

We acknowledge that this review assessed RCT articles published in 8 orthopedic journals, which may not be a representative sample of the whole orthopedic literature. Also, only RCTs allocated in 2 arms with 1 continuous primary outcome and reported power calculation were included. In addition, due to deficiencies in reported parameters of variability (SD) and uncertainty (CI_MD_), we were able to compare in only a limited number of studies the estimated and the observed values of SD and MD.

## Conclusion

Power calculations were used in most of the RCTs, but most of the studies lacked some of the essential components required by the CONSORT statement and the results required to replicate the analysis. The key parameters of data variability were also poorly reported. Low power is likely to prevail in orthopedics, but we observed good correspondence between the estimated and the observed SD of the study data among recent orthopedic RCTs. Hence, we postulate that low power is not fully responsible for the unreasonably small variability estimates in primary outcome measures. In fewer than half of the studies, the estimated MD overlapped with the CI_MD_ in primary outcome, indicating that the conclusions based on these studies are very limited. An increase in power and sample size would yield lower uncertainty of effect size and serve to mitigate this issue. Further studies are needed to investigate the interpretation of negative studies in orthopedics.

### Funding, data sharing, and potential conflicts of interests

This study had no funding. The data assessment table (as an csv file) can be obtained from the authors. The authors declare that they have no competing interests.

## Supplementary Material

Supplemental Material

## Supplementary data

The statistical codes are are available as supplementary data in the online version of this article, http://dx.doi.org/10.1080/ 17453674.2020.1755932
