# Supplementary material for: Assessing variability and uncertainty in orthopedic randomized controlled trials
Source: Acta Orthop. 2020 Apr 22;91(4):479–84. doi: 10.1080/17453674.2020.1755932 (PMC8023899; doi:10.1080/17453674.2020.1755932)
Supplement: Supplemental Material [file IORT_A_1755932_SM6468.pdf]

Supplementary data

Appendix

Variable in result	Coding
id	Name of the first author
journal	Journal abbreviation
group1	Number of patients in study group 1 in last followup
group2	Number of patients in study group 2 in last followup
power	Power calculation reported (0 = no; 1 = yes)
beta	beta error rate reported, e.g. For 80% power, beta is 1–0.8 = 0.2
alfa	alfa error rate reported for power caluclation, e.g. 5% = 0.05
es	Power outcome variable (cont=continuous, bin=binary, ni=noninferiority design, several=multiple power outcomes, generic=only standardized effect size reported)
Outcome classification based on computed or reported CI of mean difference	Based on reported confidence interval (CI) of observed mean difference (MD) in study. Statistically significnat p < 0.05 = “pos” and statistically not significant p> 0.05 = “neg”
Follow-up time point for results	Follow-up time point if available for study results. The pre-specified follow-up time point was utilized or the latest follow-up time point
Power outcome	Name of the power outcome, abbreviations used for PROMs
SD estimate	SD estimate in power calculation
MD estimate	MD estimate in power calculation
Effect size of power calculation	Effect size estimated in power calculation if SDest and MDest both reported
SD assessed	Some SD parameter accessible for power outcome from text or table (SD, SE, CI). “nr” = not reported
N1	Number of patients in study group 1 in the pre-specified or the last follow-up
N2	Number of patients in study group 2 in the pre-specified or the last follow-up
N total of trial	Total number of patients in study groups in the pre-specified or the last follow-up
Mean1	Mean of study group 1 for power outcome
Mean2	Mean of study group 2 for power outcome
SD1 outcome	SD observed for study group 1. Calculated from SE or CI when not available
SD2 outcome	SD observed for study group 2. Calculated from SE or CI when not available
SD1/SDest	Ratio of SD observed and SD estimated for study group 1
SD2/SDest	Ratio of SD observed and SD estimated for study group 2
Ln SD1/SDest	Natural logarithm of SD1/SDest ratio
Ln SD2/SDest	Natural logarithm of SD2/SDest ratio
SE1	Standard error (SE) for study group 1. Calculated from CI of mean for study group 1 if not available
SE2	Standard error (SE) for study group 2. Calculated from CI of mean for study group 2 if not available
SDrep	SD observed for total in study for power outcome. Calculated from SD1 and SD2 if not available. If study groups had no SD/SE/CI then total SD observed was calculated from CI of mean difference for primary outcome
t-value (95%)	t-value from t-distribution for 95% interval
CI low point, reported	Low point of CI of MD for primary outcome if reported
CI up-point, reported	High point of CI of MD for primary outcome if reported
CI of MD in text/table	Yes if CI of MD for primary outcome reported in text or table
CI of Means in text/table	Yes if CI of means of study groups for primary outcome reported in text or table
SDobs / SDest	Ratio of SD observed in study and SD estimated in power calculation
ln Sdobs/SDest	Natural logarithm of ratio of SD observed and SD estimated
CI low point, calculated	CI high point calculated for uncommon SD in study groups
CI up-point, calculated	CI high point calculated for uncommon SD in study groups
MSE	Mean standard error
CI up	CI high point calculated for common SD in study groups
CI low	CI high point calculated for common SD in study groups
Ci up final, CI low final	If CI of MD was reported in study, these estimates were preferred. If CI of MD was calculated, uncommon SD calculation for CI of MD was preferred
CI max	Absolute maximum point of CI of MD interval, positive or negative number
MDest not included	MD estimate in power calculation inclusion to CI of MD. (0 = included and 1 = not included)
Reason no N_SD	Reason provided for SD of power outcome not accessible.
ES MD not included	Effect size estimated in power calculation if MD estimate was not included in CI of MD
ES MD included	Effect size estimated in power calculation if MD estimate was included in CI of MD
ES MD not included NEG	Effect size estimated in power calculation if MD estimate was not included in CI of MD. In “negative” studies only
ES MD included NEG	Effect size estimated in power calculation if MD estimate was included in CI of MD. In “negative” studies only
SS MD not included neg	Sample size of study in the pre-specified or last follow-up if MD estimate was not included in CI of MD. In “negative” studies only
SS MD included neg	Sample size of study in the pre-specified or last follow-up if MD estimate was included in CI of MD. In “negative” studies only
